# Minicircle Mediated Gene Delivery to Canine and Equine Mesenchymal Stem Cells

**DOI:** 10.3390/ijms18040819

**Published:** 2017-04-12

**Authors:** Naomie Tidd, Jacob Michelsen, Bryan Hilbert, Jane C. Quinn

**Affiliations:** 1 School of Animal and Veterinary Science, Charles Sturt University, Boorooma Street, Locked Bag 588, Wagga Wagga, NSW 2678, Australia; ntidd@csu.edu.au (N.T.); jmichelsen@csu.edu.au (J.M.); bhilbert@csu.edu.au (B.H.); 2 Graham Centre for Agricultural Innovation (NSW Department of Primary Industries and Charles Sturt University), Wagga Wagga, NSW 2678, Australia

**Keywords:** canine, equine, mesenchymal stem cell, minicircle, transfection, *Sox9*

## Abstract

Gene-directed tissue repair offers the clinician, human or veterinary, the chance to enhance cartilage regeneration and repair at a molecular level. Non-viral plasmid vectors have key biosafety advantages over viral vector systems for regenerative therapies due to their episomal integration however, conventional non-viral vectors can suffer from low transfection efficiency. Our objective was to identify and validate in vitro a novel non-viral gene expression vector that could be utilized for ex vivo and in vivo delivery to stromal-derived mesenchymal stem cells (MSCs). Minicircle plasmid DNA vector containing green fluorescent protein (GFP) was generated and transfected into adipose-derived MSCs from three species: canine, equine and rodent and transfection efficiency was determined. Both canine and rat cells showed transfection efficiencies of approximately 40% using minicircle vectors with equine cells exhibiting lower transfection efficiency. A *Sox9*-expressing minicircle vector was generated and transfected into canine MSCs. Successful transfection of the minicircle-*Sox9* vector was confirmed in canine cells by Sox9 immunostaining. This study demonstrate the application and efficacy of a novel non-viral expression vector in canine and equine MSCs. Minicircle vectors have potential use in gene-directed regenerative therapies in non-rodent animal models for treatment of cartilage injury and repair.

## 1. Introduction

Joint injury and disease are debilitating in both humans and animals, often responding poorly to medical intervention. Enhancing effective cartilage regeneration could hold the key to improving outcomes for human and veterinary patients alike.

A number of species have been examined in terms of their ability to generate cartilage matrix from adult-derived mesenchymal stem cells (MSCs) including humans and small animals such as rabbits [1]. Large animal species have included cattle [2] and horses [3,4]. However the differentiation potential of adipose-derived MSCs have been less thoroughly characterized in canines and equines than in other species [5,6] with canine MSCs shown to be particularly problematic in regard to induction of chondrogenic differentiation [7,8].

Induction of in vitro chondrogenesis of MSCs from non-traditional laboratory species requires refinement. One approach to improve this process may be through gene-directed approaches, thereby enhancing the in vivo differentiation of host-derived mesenchymal stem cells into healthy chondrocytes, enhancing their integration into damaged tissues [9]. Genetic modification of MSCs with the high-mobility-group (HMG) domain transcription factor *Sox9* [10,11,12] alone or in combination with Sox5 and Sox6 (Sox trio) [13,14] has been trialed as an approach to improve chondrogenic outcomes for MSC-mediated regenerative therapies, with encouraging results. These studies have shown an increased synthesis of extracellular matrix proteins associated with cartilage formation and an up-regulation in the expression of cartilage-specific genes such as type 2 collagen (COL2A1) and aggrecan compared to controls, in both human and rodent cell lines [10,11,15]. However, there are significant biosafety issues to be overcome before this approach can be applied in a clinical setting, not least of which is the use of genetically modified material for transplantation.

Both viral and non-viral vectors have been used for gene delivery to somatic cells. Non-viral plasmid vectors have key safety advantages over viral vectors due to their reduced immunogenicity, lack of potential for disease transmission [16] and a low risk of integration into the host genome [17]. However, the large size of the conventional plasmid backbone can restrict vector diffusion [18] resulting in low transfection efficiency, whilst bacterial elements may induce significant alternative immunological activation in the recipient [19,20,21]. In addition, presence of exogenous genomic sequences, such as antibiotic resistance genes, has become unacceptable for gene therapy due to the increased risk of their subsequent transfer to the environment [22]. Together these obstacles make the need for the identification of safe and effective expression vectors for use in gene-directed regenerative medicine all the more compelling.

Minicircle vectors are small, supercoiled plasmids lacking prokaryotic backbone sequences, such as those encoding bacterial origin of replication and antibiotic resistance genes in present traditional plasmid vectors [23]. Minicircle plasmids have demonstrated, in vitro and in vivo, to have higher transfection efficiency and an ability to initiate higher levels of transgene expression than traditional plasmids [24,25]. Minicircles have been used in a number of recent preliminary trials in rodents where they have induced robust expression of target genes in the heart [26], and liver [25,27,28]. These encouraging developments suggest that minicircle vectors may be suitable vectors for ex vivo modification of MSCs for cartilage regeneration. Currently, minicircle vectors are amongst the most promising tools currently available for use in regenerative therapies in both man and animals.

Despite horses and larger breeds of dogs representing suitable animal model systems to study comparative regenerative approaches in humans due to their size and weight-bearing of their limbs [29,30], there is paucity in literature regarding genetic modification of MSCs derived from these species. To address this issue, we assessed minicircle plasmid-mediated transfection of MSCs in two non-rodent species, canines and equines. An engineered minicircle plasmid from a parental plasmid encoding the transcription factor *Sox9* was also assessed for its capacity to induce *Sox9* in the target cells. Together, the findings from these investigations identify Minicircle vectors as an effective tool for gene delivery in two species of veterinary and comparative clinical importance.

## 2. Results

### 2.1. Isolation and Characterisation of Canine and Equine Mesenchymal Stem Cells

**Figure 1:**
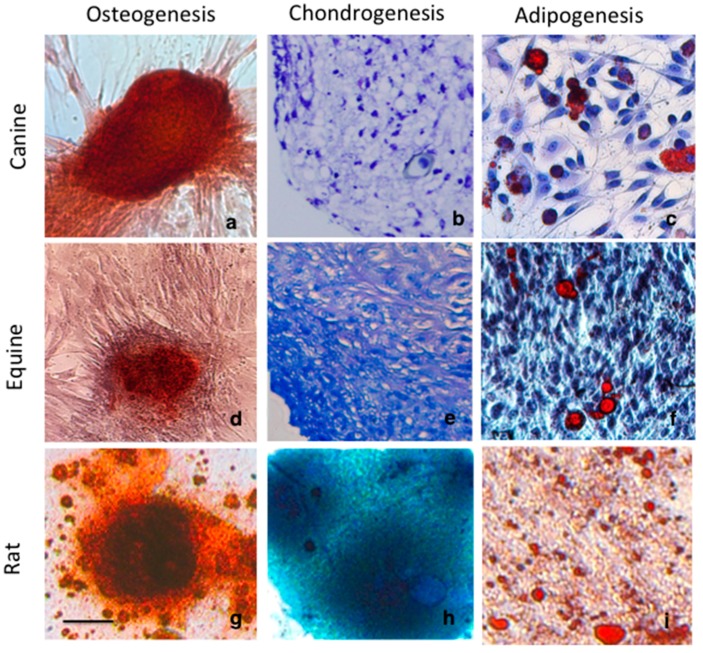
Differentiation assays of canine, equine and rat mesenchymal stem cells showing (**a**,**d**,**g**) positive osteogenesis by staining of mineral accumulation within nodules with Alizarin Red S. (**b**,**e,h**) chondrogenic differentiation is identified by glycosaminoglycan accumulation throughout the cell pellet, following staining with toluidine blue or alcian blue of rat MSCs and (**c**,**f,i**) Adipogenic differentiation is confirmed Oil Red O staining. Scale = 100 µm.

Tri-lineage differentiation (adipose tissue, bone, cartilage) was confirmed in all MSC lines used for this study [3,31,32]. Canine and equine MSCs were isolated from adipose tissue and cultured in vitro. Rat MSCs were included as a traditional species comparison. Rat MSCs were produced by culture of cells isolated from juvenile rat femur. Using standard differentiation protocols, we differentiated MSCs to an osteogenic phenotype as shown by nodule formation and positive staining for mineralization (Figure 1a,d,g). Equine, canine and rat MSCs were able to undergo adipocyte differentiation, confirmed by the observation of lipid droplets by Oil Red O staining (Figure 1c,f,i).

Chondrogenic induction produced cells with characteristic chondrocyte morphology expressing glycosaminoglycans (GAG) as identified by toluidine blue staining in canine and equine MSCs (Figure 1c,f) and alcian blue staining of proteoglycans in rat MSCs (Figure 1h). 

### 2.2. Further Chondrogenic Characterisation of Equine and Canine MSCs

Comparatively, equine MSCS showed more extensive GAG-expressing cell accumulation following chondrogenic induction than canine MSCs (Figure 1b,e). Canine and equine MSCs cultured in three-dimensional micromass pellet [31,33] appeared initially as a flattened pellet at the base of the tube, which became spherical after several days. After 21 days, pellets of equine MSCs changed from white to an opaque colour with a shiny appearance and increased in size dramatically. Canine MSCs grown in induction media, showed little change in size throughout the incubation period and remained a comparable size to the non-induced control pellet. 

**Figure 2:**
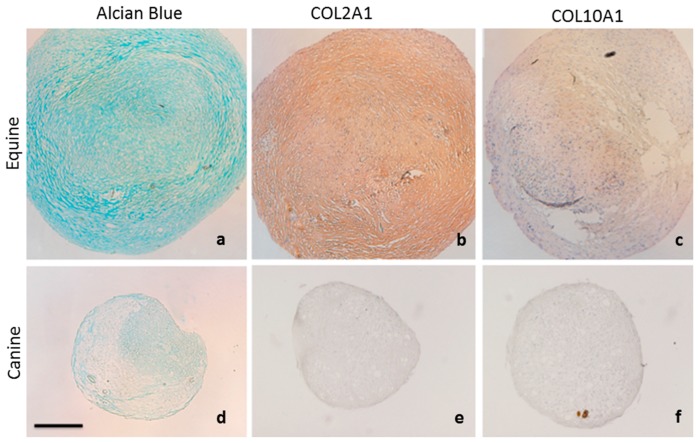
Chondrogenic characterisation of equine and canine MSCs. (**a**) Equine MSCs show extensive proteoglycan accumulation following staining with Alcian blue. (**b**) Immunohistochemistry demonstrates uniform distribution of COL2A1 throughout equine MSC pellet (**c**) Minimal expression of COL10A1 is observed in equine MSCs after three weeks in culture. Canine MSCs show poor expression of (**d**) proteoglycans (**e**) COL2A1and (**f**) COL10A1. Scale = 200 µm.

Equine and canine MSCs were further characterised for the chondrogenic markers acidic proteoglycans and type 2 collagen (COL2A1) as well as the hypertrophic marker type 10 collagen X (COL10A1) (Figure 2). Micromass pellets of equine MSCs showed extensive extracellular matrix deposition which stained positive for the presence of acidic proteoglycans using alcian blue (Figure 2a) Immunohistochemistry of equine MSC pellets also showed uniform expression of the hyaline cartilage marker COL2A1 (Figure 2b), but very little expression of COL10A1, suggesting that equine MSC pellets were not transforming to a hypertrophic phenotype after three weeks in culture. In comparison, canine MSCs showed very little deposition of proteoglycans or COL2A1 (Figure 2d,e).

Although little expression of COL10A1 was observed (Figure 2f), the poor expression of chondrogenic markers glycosaminoglycan, proteoglycan and COL2A1 suggests inferior chondrogenic induction of canine MSCs in comparison to equine MSCs.

### 2.3. Transfection Using a Minicircle Vector Shows Increased Transgene Expression Compared to Parental Plasmids in Rat, Canine and Equine MSCs

**Figure 3:**
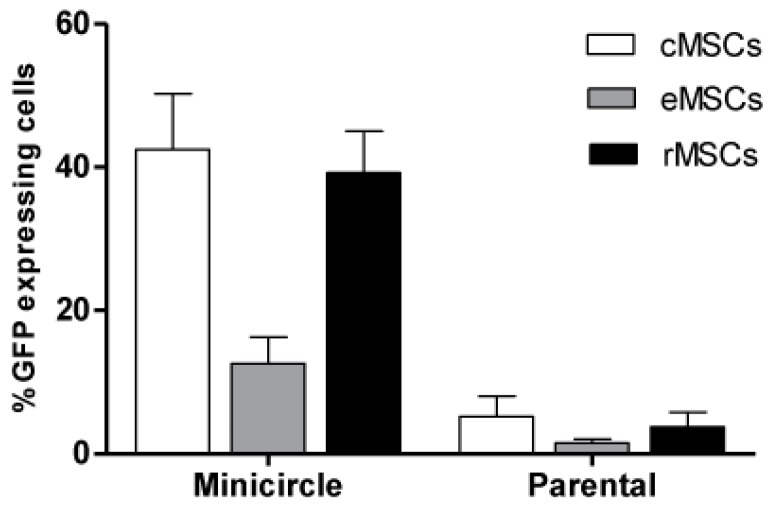
Percentage of green fluorescent protein (GFP) expressing cells after transfection using minicircle and parental plasmids in rat, canine and equine MSCs. Canine and rat MSCs showed similar transfection efficiencies with minicircle and parental plasmid, whilst equine MSCs showed the least. Data presented as mean ± SE.

Transfection efficiency in rat, equine and canine MSCs was compared between the 7 kb parental minicircle plasmid (pMC.CAG) and the smaller 3 kb minicircle plasmid (MC.CAG), as determined by percentage of green fluorescent protein (GFP) expression following transfection (Figure 3). A significant difference was observed between the percentages of GFP expressing cells after transfection with minicircle plasmid when compared to parental plasmid (*p* < 0.001). Canine and rodent MSCs displayed similar percentages of fluorescent cells after transfection with pMC.CAG (canine MSC: 5.2% ± 2.8%; rat MSC: 3.8% ± 2%) and MC.CAG (canine MSC: 43.05% ± 7.6%: rat MSC: 39.07% ± 5.8%). Equine MSCs showed the lowest percentage of fluorescent cells in comparison to the other species following transfection with both vectors (Figure 3). Percentages of GFP-expressing equine MSCs were 1.5% ± 0.54% following transfection with pMC.CAG and 13.00% ± 3.6% with MC.CAG transfection.

### 2.4. Minicircle Plasmid Demonstrates Similar GFP Expression Intensity in Rat, Canine and Equine MSCs and Is Diluted with Passage

**Figure 4:**
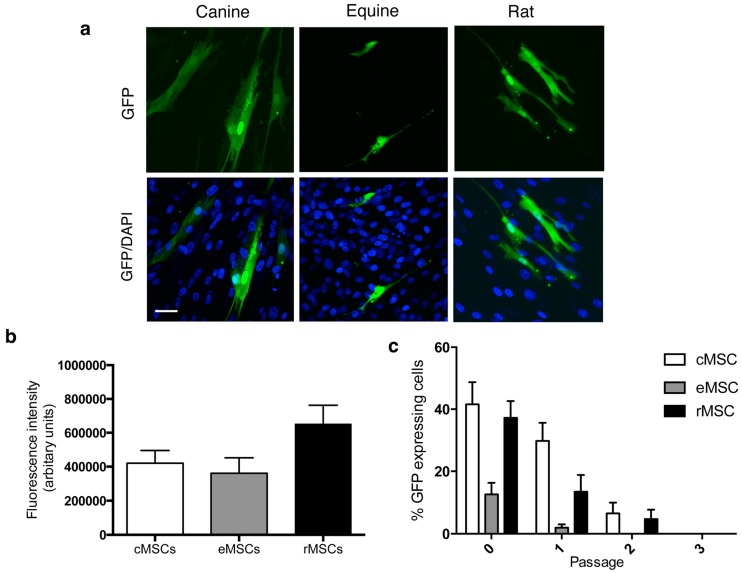
(**a**) GFP expression was assessed in MSCs transfected with MC.CAG. (**b**) The average relative fluorescent intensity was similar between all three cell lines examined. (**c**) GFP expression decreased with passage and dilution of MC.CAG, with no GFP expression visible at passage 2 for equine MSCs and passage 3 for canine and rat MSCs. Data presented as mean ± SE. (*n* = 25 cells from three independent experiments). Scale bar = 50 μm.

MSCs transfected with MC.CAG were assessed for GFP expression frequency (Figure 4a) and intensity (Figure 4b). The average relative GFP expression intensity was similar between all three cell lines examined, with no significant difference observed (*p* = 0.07). Following passage, the percentage of cells expressing GFP declined. After passage 1, 1.9% ± 0.4% of equine MSCs showed GFP expression in cells transfected with MC.CAG, with no GFP observed in equine MSCs after passage 2 (Figure 4c). After passage 2, ten days following initial transfection with MC.CAG, 6.5% ± 1.2% and 4.7% ± 1.0% cells expressed GFP in canine and rat MSC cultures respectively (Figure 4c). GFP signal could not be identified after passage 3.

### 2.5. Minicircle Vector-Driven Expression of Sox9 in Canine MSCs

**Figure 5:**
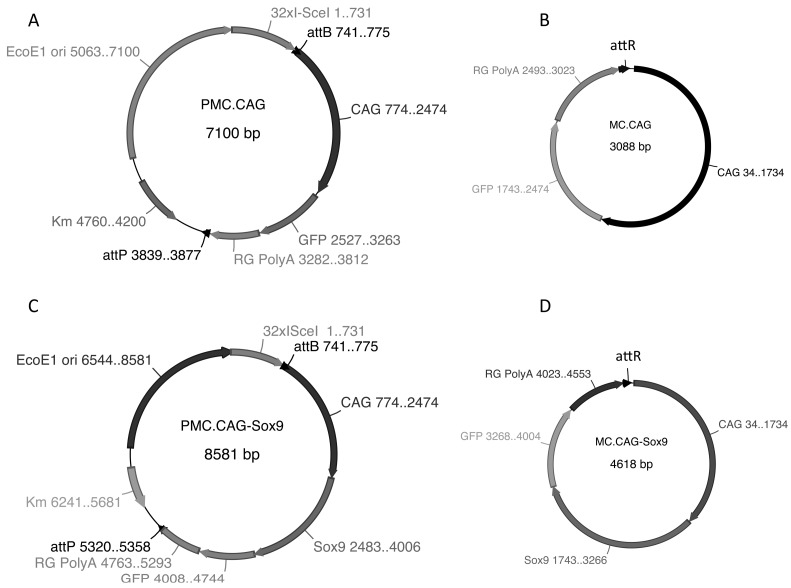
(**A**) Parental plasmid pMC-CAG-MCS-GFP-Rabbit globin PolyA (pMC.CAG) contains CMV early enhancer/chicken beta actin promoter (CAG), multiple cloning site (MCS), green fluorescent protein (GFP) and polyadenylation signal from rabbit globin (RG PolyA). (**C**) pMC-CAG-*Sox9*-GFP-Rabbit globin PolyA (pMC.CAG-*Sox9*) is identical to pMC.CAG with murine *Sox9* clone between EcoR1 and Bam H1 restriction enzyme sites. (**B**,**D**) Minicircle plasmids are formed through molecular recombination of att sites and degradation of the parental plasmid backbone following arabinose induction of producer *Escherichia coli* (*E. coli*) strain.

**Figure 6:**
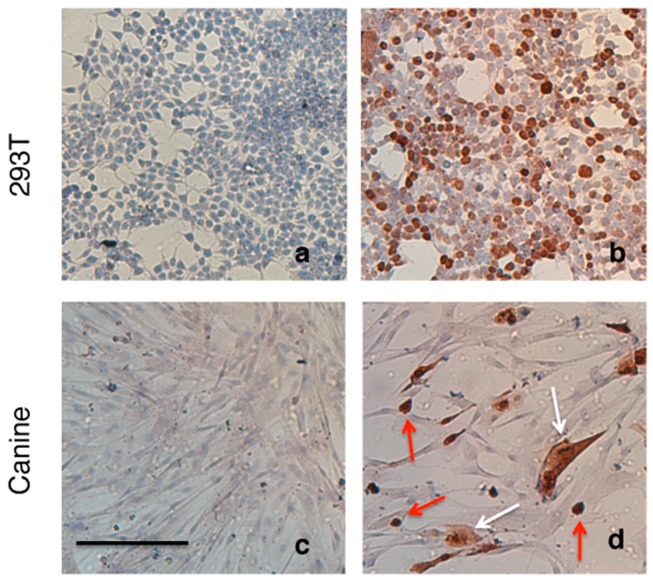
*Sox9* immunostaining of canine and HEK293T cell monolayers. (**a**) HEK293T and (**c**) canine MSC in the absence of transfection with MC-CAG.Sox9 vector, no endogenous Sox9 expression is apparent in these culture. (**b**) HEK293T and (**d**) canine MSCs MC-CAG.Sox9 vector *Sox9* transfected cells showing positive staining for Sox9 protein. Nuclear localization can be observed Sox9 (red arrows), while others show nuclear and cytoplasmic localisation of Sox9 (white arrows). Scale = 200 µm.

Canine MSCs and HEK293T cells were transfected with MC.CAG-*Sox9* minicircle plasmid DNA (Figure 5) using Lipofectamine^®^ LTX (Thermo Fisher Scientific, Walham, MA, USA). Strong expression of *Sox9* protein was observed within the nucleus and cytoplasm of both transfected cMSCs (Figure 6d) and HEK293T cells (Figure 6b) using immunohistochemistry (Figure 6). Canine MSCs transfected with MC.CAG-*Sox9* minicircle plasmid displayed 25.58 ± 6.69% (s.d.) positive expression of *Sox9* protein.

### 2.6. Discussion

Effective gene therapy requires a safe and efficient gene delivery method, coupled with sustained or controlled expression of the gene of interest. Non-viral vectors have safety advantages over viral vectors, but their clinical use in regenerative therapies is limited by poor transfection efficiencies, host immune responses and gene silencing [26].

Minicircle plasmids are small, supercoiled plasmids devoid of prokaryotic backbone sequences. Due to their non-microbial origin of replication, and lack of antibiotic resistance genes, they offer a safer alternative than viral or traditional plasmid vectors for clinical approaches [23]. They have also been shown in vitro and in vivo to improve the extent and duration of transgene expression [20,24,34,35]. In this study we were able to show that minicircle vectors showed good efficacy over traditional vector systems in mesenchymal stem cells isolated from two non-human species, dogs and horses, species which can provide suitable model systems for human clinical trials as well as for veterinary clinical applications. Results of this study identified that by using a minicircle vector we were able to show greater than 35% GFP-expressing (transfected) cells isolated from canine hosts, values comparable to that observed in rodent cells, with similar levels of GFP expression across all cell lines examined. Equine MSCs showed a reduced transfection efficiency with minicircle vector suggesting that more studies are required to confirm optimal utilisation of this delivery mechanism in equine-derived cells. The reasons for this relative transfection inefficiency in equine cells is unclear, but could be related to age of donor or unknown factors affecting vector uptake in cells of this species. 

Equine MSCs showed a greater chondrogenic capacity than canine MSCs with more extensive extracellular matrix deposition and production of glycosaminonglycans, proteoglycans and Col2 (Figure 1 and Figure 2). Chondrogenic differentiation of canine MSCs, particularly, has been shown to be problematic [7,8] and has often omitted from published characterization and differentiation assays [36,37,38,39]. Although reasons for this poor chondrogenic differentiation is unclear, cell senescence and the sudden change in culture conditions to high cell density pellet culture with low nutrient and low oxygen [40] have been shown to influence the ability of some cell types to proliferate and/or differentiate.

One approach to address poor chondrogenesic outcomes in canine MSCs may be through genetic modification using a non-viral vector such as the minicircle. The transcription factor *Sox9* is known to play a significant role during chondrogenesis [41,42,43,44,45] and represents an ideal target gene to potentially enhance chondrogenesis of canine MSCs. 

Lacking bacterial elements, minicircle plasmids have been shown to have a greater tendency to resist integration into the host genome than conventional plasmids [19]. Results of this study show that GFP expression decreased with passage, suggesting that minicircle plasmid was successfully diluted with each division of canine, equine and rat MSCs (Figure 4).

The minicircle plasmid is a 3 kb vector, which is small in size compared to traditional vectors systems, a factor which is suggested to increases its relative transfection efficiency. In this study, the minicircle vectors displayed a higher transfection rate across all the three species examined than the larger 7 kb parental plasmids, giving a 14-fold increase in proportion of GFP-expression cells post transfection. Part of this improvement might be attributed to weight-to-weight transfection of parental to minicircle plasmid DNA [46]. It is estimated from these experiments that a 2.2-fold increase in the number of GFP-expression cassettes are transferred following minicircle plasmid transfection in comparison to parental plasmid transfection. However, a proportional increase in GFP expression in those transfected cells cannot be assumed. A high number of GFP molecules is required per cell, (approximately 10^5^), before GFP becomes detectable [47]. A 2.2-fold increase in the number of GFP molecules per cell may not be sufficient to reach this minimum threshold. Minicircle plasmids have been shown to give higher levels of transgene expression even when transfected at the same molar concentration [34,48,49]. Therefore this difference in expression between minicircle and traditional vectors in this study cannot be attributed entirely to concentration and suggests minicircle vectors to have higher transfection efficiency than standard parental plasmids in canine, equine and rat MSCs. 

Transfected plasmid DNA must first traverse the cell cytoplasm and then enter the nucleus prior to transcription and then subsequent expression of the target protein [50]. Vector complex size is known to affect the mechanism of cell internalization [51,52] and plays an important role in endocytosis and migration of the expression package to the nucleus [23], with cytoplasmic mobility and nuclear entry inversely proportional to the size of the plasmid DNA [53]. Since minicircle plasmids form smaller complexes with cationic lipids, such as Lipofectamine, these smaller minicircle complexes may interact more efficiently with the cell membrane, leading to an improved cellular uptake [48], and enhanced intracellular trafficking and nuclear entry [24]. Minicircle plasmid has been previously confirmed as a superior expression vector in comparison to parental plasmid in rodent and human cell lines [24,34,47,49]. The result of this study confirms efficacy in canine and rat MSCs and suggests that it may be a useful vector for in vitro and in vivo gene transfer in these model systems.

Equine MSCs showed the lowest percentages of GFP expressing cells of all species examined (Figure 2). Jia and colleagues (2010) observed similar results in human cell lines where only 10.8% ± 1.7% of cells showed GFP expression following transfection of a minicircle vector in human adipose-derived stem cells. Suitable concentration of transfected cells was only achieved via fluorescence activated cell sorting (FACS) [54]. The mechanisms underlying poor transfection efficiency of equine cells may give insights into the same limitations in human tissue derived MSC cell populations.

In mammalian cells the mechanisms of cellular attachment and internalisation of DNA-lipid complexes following transfection, has been shown to be cell line-dependant [51,55]. Studies investigating transfection efficiencies of human cervical carcinoma (HeLa) cells, African green monkey kidney fibroblast-like cell line (COS-7), Chinese hamster ovary (CHO) and HEK293T cell lines have shown differences in their internalisation pathways, which have resulted in variable transfection efficiency [51,55]. It is possible that equine MSCs exhibit a similar effect on the subcelluar machinery required for effective internalisation, compared to canine and rat MSCs, affecting cellular uptake and intracellular trafficking of plasmid motifs and therefore transfection efficiency. Further research is needed to compare other methods of non-viral vector delivery, such as nucleofection, in these cell lines that may begin to answer some of these questions. 

Construction and validation of a *Sox9*-expressing minicircle vector was achieved in this study. *Sox9* is involved at all stages of chondrocyte development and remains expressed in chondrocytes throughout adult life [42,45,56]. *Sox9* has been also hypothesised to be sufficient to drive MSCs to a chondrogenic fate, an outcome with potential usefulness for gene directed regenerative therapies for cartilage injury and repair. Generation of a *Sox9* expressing minicircle vector was achieved in this study and successfully demonstrated to deliver Sox9 expression with high efficiency in canine MSCs (Figure 5). 

## 3. Materials and Methods

All experiments involving animals were undertaken with approval from Charles Sturt University Animal Care and Ethics Committee Wagga Wagga, New South Wales, Australia and in accordance with the Australian code of practice for the care and use of animals for scientific purposes. All chemicals and reagents were sourced from Sigma-Aldrich (St. Louis, MO, USA) unless otherwise stated.

### 3.1. Vectors

Minicircle plasmids MC.CAG and MC.CAG-Sox9 were produced in arabinose-inducible producer strain *E. coli* ZYCY10P3S2T (System Biosciences, Palo Alto, CA, USA) expresses genes for PhiC31 integrase and I-Sce1 endonuclease. 

Plasmid pMC-CAG-MCS-GFP-Rabbit globin PolyA (pMC.CAG) containing the human cytomegalovirus (CMV) early enhancer/chicken beta actin promoter (CAG), multiple cloning site (MCS), green fluorescent protein (GFP) was used as a parental plasmid and for generation of *Sox9* expressing vector (Figure 5).

The construction of parental plasmid containing a CAG-Sox9 expression sequence (pMC.CAG-*Sox9*) was achieved by cloning a 1.5 kb murine *Sox9* cDNA into the pMC.CAG vector. *Sox9* cDNA sequences were initially amplified from template DNA by PCR using Phusion high-fidelity DNA polymerase (New England Biolabs, Ipswich, MA, USA) before insertion into the parental pMC.CAG plasmid by cold fusion cloning (System Biosciences, CA, USA). Orientation of the *Sox9* fragment was verified by sequence analysis.

### 3.2. Plasmid Production and Purification

Parental plasmids pMC.CAG and pMC.CAG-Sox9 were transformed into ZYCY10P3S2T *E. coli* competent cells, clonal colonies were selected for plasmid isolation. The remaining colonies were combined with minicircle induction containing 8 mL 1M NaOH and 200 µL 20% l-arabinose, before incubation at 30 °C with shaking for at least 5 h to allow adequate *att* recombination and parental plasmid backbone degradation. Minicircle and parental plasmid DNA was isolated from the separate pellets using Qiagen High speed Maxiprep plasmid isolation kit (Qiagen, Valencia, CA, USA). The purity of all plasmids was verified by absorbance readings at 260 and 280 nm as well as restriction enzyme digestion and gel electrophoresis. 

### 3.3. Isolation and Subculture of Canine and Equine Adipose Tissue-Derived MSCs

Derivation of canine adipose-derived MSCs (cMSC) was achieved by dissection of adipose tissue from the discarded falciform ligament during routine ovari-hysterectomy procedures of six female dogs, ranging in age from six months to seven years. Adipose-derived equine MSCs were isolated from subcutaneous adipose tissue collected aseptically from above the dorsal gluteal muscle from a total of five horses according to published protocols. Pooled populations of equine (*n* = 5), canine (*n* = 4) were used for all experiments.

After isolation by fractionation, MSCs of both species were maintained in complete Dulbecco’s Modified Eagle Medium (DMEM) expansion media (Life Technologies, Carlsbad, CA, USA) containing 10% foetal bovine serum (FBS) and 1% penicillin-streptomycin-glutamine (Life Technologies, Carlsbad, CA, USA) at 37 °C, 5% CO_2_ in a humidified incubator. MSCs were expanded as required use in differentiation or transfection assays. Canine MSCs (cMSCs) were used at passage 5 or less and equine MSCs were used at passage 9 or less for all experiments.

### 3.4. Adipogenic Induction of Canine and Equine MSCs

For adipogenic induction, MSCs were plated in triplicate into 24 well plates at 6 × 10^4^ cells per well. The cells were expanded until 100% confluent in complete DMEM expansion media and incubated at 37 °C with 5% CO_2_. Adipogenic induction was carried out using adipogenic induction media containing 15% rabbit serum, 1 µM dexamethasone, 0.5 mM IBMX inhibitor, 10 µg/mL insulin and 100 µM indomethacin according to published protocols [43]. Following adipogenic induction, cells were fixed in 4% paraformaldehyde before identification of oil droplets by Oil Red O staining (Sigma-Aldrich). 

### 3.5. Osteogenic Induction

MSCs were expanded in complete media until 100% confluent. Osteogenic induction was carried out using media containing 10% fetal bovine serum (FBS), 0.2 mM Ascorbic acid 2-phosphate, 0.1 µM dexamethasone, and 10 mM β glycerol 2-phosphate according to published protocols [24]. Cells were maintained in the induction protocol for 17 days after which cells were stained for mineral deposition using Alizarin Red S (Sigma-Aldrich).

### 3.6. Chondrogenic Induction

Canine and equine MSCs were induced to form chondrocytes by three-dimensional micromass pellet culture [25,44]. Briefly, cell suspensions containing 2.5 × 10^5^ cells were pelleted by centrifugation and chondrogenic induction initiated using 1% ITS, 0.1 mM ascorbate 2-phosphate, 0.1 µM dexamethasone, 350 µM l-proline and 10 ng/mL Transforming Growth Factor-β3 (TGF-β3, Peprotech, Rocky Hill, NJ, USA). Pellets were incubated at 37 °C at 5% CO_2_ for a maximum of 21 days. Pellets were fixed overnight in 10% neutral buffered formalin before processing, sectioning and staining for toluidine blue and alcian blue for presence of glycosaminoglycans and proteoglycans [26]. 

### 3.7. COL2A1 and COL10A1 Immunohistochemistry

Antigen retrieval of 4 µm sections was performed in Envision Flex high pH buffer (DAKO, Glostrup, Denmark) using DAKO pre-treatment module (PT link). Sections were blocked in blocking buffer for 1 h at room temperature followed by peroxidase block for 15 min. Primary antibody COL2 (Abcam Ab34712) or COL10 (Ab58632) was applied and incubated at room temperature for 1 h. After washing rabbit/mouse secondary antibody (DAKO) was applied and allowed to incubate for 30 min at room temperature. The sections were then washed three times in TBS buffer before applying the Envison DAB chromagen (DAKO).

### 3.8. Transfection of Rat, Canine and Equine MSCs

In addition to MSC lines derived as described above, bone marrow derived rat MSCs (rMSC) and human embryonic kidney (HEK) 293T cells were used as cell line transfection controls. For transfection with minicircle vectors, MSCs were seeded into 24 well plates at a rate of 0.8 × 10^5^ cells per well and transfected when 90% confluent. Transfections were performed in triplicate over three independent experiments, using an optimised rate of 0.5 µg DNA per well with 0.5 µL Plus™ reagent and 2 µL transfection reagent Lipofectamine^®^ LTX (Thermo Fisher Scientific) in combination with Opti-MEM^®^ reduced serum medium (Thermo Fisher Scientific). 

### 3.9. Comparison of Transfection Efficiency between Parental and Minicircle Plasmids

Expression efficiency was compared between minicircle plasmids (3 kb) and their larger conventional-type parental plasmids (7 kb), by analysis of percentage of GFP expressing cells after transfection of the same concentration of plasmid DNA, weight: weight. It was not desirable to compare plasmids using transfection of DNA at the same molar ratio, as this would require the addition of stuffer or carrier DNA to maintain the correct linear ratio of Lipofectamine^®^ to DNA between the two constructs [16,32]. 

### 3.10. Passage of MSCs Transfected with MC-CAG

MSC monolayers were disrupted using trypsin digestion (0.25%) between 48–72 h post-transfection before redistribution in fresh complete DMEM. Half of the cells from the monolayer were analysed for percentage of GFP expressing cells using fluorescent microscopy. Images of transfected cells were taken using fluorescent confocal microscopy and quantification of the image fluorescent intensity was performed with ImageJ™ software (US National Institutes of Health, Bethesda, MD, USA).

The remaining cells from the monolayer were plated into a new 24 well plate and fresh media added. After a further 4 days, the monolayer was again dislodged from the base of the well. Half of the cell monolayer re-plated for a further passage and the remainder analysed for GFP expression again. This was repeated once more making a total of three passages and four GFP expression analyses following transfection.

### 3.11. Transfection of Canine MSCs with MC.CAG-Sox9

Canine MSCs cells were seeded into 24 well plates at a rate of 0.8 × 10^5^ cells per well and transfected when 90% confluent. HEK293T transfection control cells were seeded at a rate of 0.2 × 10^5^ cells per well and were transfected at 90% confluency. MC.CAG-Sox9 plasmid DNA was transfected into all cell monolayers at a concentration of 0.5 µg per well, using 0.5 µL Plus^®^ reagent and 2 µL of Lipofectamine^®^ LTX. After overnight incubation, fresh media was added to the cell monolayers and plates were incubated for a further 3 days before immunostaining for visualisation of *Sox9* protein production. The proportion of canine MSCs displaying positive expression of *Sox9* protein were assessed with ImageJ software.

### 3.12. Immunohistochemistry

Monolayers of transfected cells were fixed in 3% paraformaldehyde (Sigma-Aldrich) before washing processing for immunostaining. Routine immunohistochemistry was undertaken using a rabbit anti-*Sox9* antibody (Santa Cruz Biotechnology, Santa Cruz, CA, USA) (1:50 dilution) before visualisation of *Sox9* reactivity using secondary amplification with EnVison™ Flex rabbit/mouse linker + DAB chromagen (DAKO, Glostrup, Denmark). Slides were dehydrated and mounted for visualisation by standard light microscopy.

### 3.13. Statistical Design and Analysis

To compare the efficacy of parental and minicircle plasmids in different species, percentage of cells expression GFP was examined and quantitated. Statistical analysis used a binomial generalised mixed linear model with fixed effects of animal*plasmid to interrogate the data. Analyses were performed using Genstat™ version 18.02.0.18409 software (VSN International Ltd., Hemel Hempstead, UK).

## 4. Conclusions

In conclusion, the results of this study highlight the efficacy of the minicircle plasmid as an expression vector with superior transfection efficiency and transgene expression over traditional plasmid vectors in adipose derived MSCs in non-human species. Paired with a suitable target gene, such as *Sox9*, minicircle vectors are simple to produce and may become accepted as potential gene therapy tools for the manipulation of MSCs for cartilage regeneration and repair in the future.
